# MIAMI‐AD (Methylation in Aging and Methylation in AD): an integrative knowledgebase that facilitates explorations of DNA methylation across sex, aging, and Alzheimer’s disease

**DOI:** 10.1002/alz.091111

**Published:** 2025-01-03

**Authors:** Lily Wang, David Lukacsovich, Deirdre O'Shea, Hanchen Huang, Wei Zhang, Juan I Young, X. Steven Chen, Sven‐Thorsten Dietrich, Brian W. Kunkle, Eden R. Martin

**Affiliations:** ^1^ John P. Hussman Institute for Human Genomics, University of Miami Miller School of Medicine, Miami, FL USA; ^2^ University of Miami Miller School of Medicine, Miami, FL USA; ^3^ University of Miami Miller School of Medicine, Boca Raton, FL USA; ^4^ John P. Hussman Institute for Human Genomics, Miller School of Medicine, Miami, FL USA

## Abstract

**Background:**

Alzheimer’s disease (AD) is a common neurodegenerative disorder with a significant impact on aging populations. DNA methylation (DNAm) alterations have been implicated in both the aging processes and the development of AD. Given that AD affects more women than men, it is also important to explore DNAm changes that occur in each sex.

**Method:**

We created the MIAMI‐AD, a comprehensive database containing manually curated summary statistics from 97 published tables in 37 studies, all of which included at least 100 participants. There are four main sections: Genome‐wide Query, Gene Query, CpG Query, and Epigenetic Clock Query.

**Result:**

MIAMI‐AD enables easy browsing, querying, and downloading DNAm associations at multiple levels – at individual CpG, gene, genomic regions, or genome‐wide, in one or multiple studies. Moreover, it also offers tools to perform integrative analyses, such as comparing DNAm associations across different phenotypes or tissues, as well as interactive visualizations. Using several use case examples, we demonstrated that MIAMI‐AD facilitates our understanding of age‐associated CpGs in AD and in revealing sex‐specific DNAm differences in AD.

**Conclusion:**

MIAMI‐AD (https://miami‐ad.org/) facilitates integrative explorations to better understand the interplay between DNAm across aging, sex, and AD. This open‐access resource is freely available to the research community, and all the underlying data can be downloaded.

